# Burden-of-Illness Associated with Bleeding-Related Hospitalizations in Atrial Fibrillation Patients: Findings from the Nationwide Readmission Database

**DOI:** 10.1055/s-0040-1716549

**Published:** 2020-09-11

**Authors:** Benjamin Miao, Monique Miller, Belinda Lovelace, Anne Beaubrun, Kelly McNeil-Posey, Mark J. Alberts, William Frank Peacock, Olivia S. Costa, Charles Michael White, Craig I. Coleman

**Affiliations:** 1 Department of Pharmacy Practice, University of Connecticut School of Pharmacy, Storrs, Connecticut, United States; 2 Evidence-Based Practice Center, Hartford Hospital, Hartford, Connecticut, United States; 3 Health Economics and Outcomes, Portola Pharmaceuticals, San Francisco, California, United States; 4 Department of Neurology, Hartford Hospital, Hartford, Connecticut, United States; 5 Department of Emergency Medicine, Baylor College of Medicine, Houston, Texas, United States

**Keywords:** hemorrhage, bleeding, atrial fibrillation, outcomes, burden-of-illness

## Abstract

**Introduction** A paucity of contemporary data examining bleeding-related hospitalization outcomes in atrial fibrillation (AF) patients exists.

**Methods** Adults in the Nationwide Readmissions Database (January 2016–November 2016) with AF and hospitalized for intracranial hemorrhage (ICH), gastrointestinal, genitourinary, or other bleeding were identified. Association between bleed types and outcomes were assessed using multivariable regression (gastrointestinal defined as referent) and reported as crude incidences and adjusted odds ratios (ORs) or mean differences with 95% confidence intervals (CIs).

**Results** In total, 196,878 index bleeding-related hospitalizations were identified in this AF cohort (CHA2DS2VASc score ≥2 in 95.1%), with 70.8% classified as gastrointestinal. The overall incidences of in-hospital mortality, need for post-discharge out-of-home care, and 30-day readmission were 4.9, 50.8, and 18.2%, respectively. Multivariable regression suggested traumatic and nontraumatic ICHs were associated with higher odds of in-hospital mortality (OR = 3.99, 95% CI = 3.79, 4.19; OR = 13.09, 95% CI = 12.24, 13.99) and need for post-discharge out-of-home care (OR = 2.92, 95% CI = 2.83, 3.01; OR = 2.74, 95% CI = 2.59, 2.90), and increases in mean index hospitalization length-of-stay (8.31 days, 95% CI = 8.03, 8.60, 6.27 days, 95% CI = 5.97, 6.57) versus gastrointestinal bleeding. Genitourinary and other bleeds were associated with lower mortality (OR = 0.37, 95% CI = 0.25, 0.55; OR = 0.59, 95% CI = 0.53, 0.64) and reduced length-of-stays (−2.84 days, 95% CI =  − 2.91, −2.76; −2.06 days, 95% CI =  − 2.11, −2.01) versus gastrointestinal bleeding. Genitourinary bleeds were also associated with a reduced need for post-discharge out-of-home care (OR = 0.86, 95% CI = 0.77, 0.97).

**Conclusion** The burden of bleeding-related hospitalizations was notably driven by relatively rare but severe and life-threatening ICH, and less morbid but more frequent gastrointestinal bleeding. There is need for continued research on bleeding risk factors and mitigation techniques to avoid bleeding-related patient hospitalizations.

## Introduction

Oral anticoagulants can prevent most ischemic strokes in patients with atrial fibrillation (AF); however, fear of severe or life-threatening (and even minor) bleeds, a perceived high risk of bleeding on oral anticoagulation in frail or higher fall risk patients and the effort required to monitor oral anticoagulants are among the most cited reasons for their persistent underutilization. While the absolute risk is low, oral anticoagulants remain a common cause of bleeding-related hospitalizations. 1 2 3 4 5 In one real-world study of patients with AF who were treated with oral factor Xa inhibitors, those who were hospitalized with a major bleed of any type were found to have mean hospital stays exceeding 5 days and inpatient mortality rates of approximately 3%, though mortality risk was notably higher for patients with intracranial hemorrhage (ICH) (approximately 14%). 4

There is a scarcity of nationally representative data describing the incidence and burden-of-illness of bleeding-related hospitalizations in AF patients, both overall and by bleed subtype. The availability of contemporary data on this topic would allow clinicians and other decision-makers to better understand the absolute and relative risks of detrimental outcomes across various bleeding-related hospitalization types. Thus, this study sought to evaluate incidence rates and subsequent outcomes associated with bleeding-related hospitalizations, overall and by bleeding subtype, among patients with AF. Though the dataset used for this analysis does not contain data on anticoagulant use, we limited the present analysis to AF patients as they are considered higher risk for thrombosis and should be treated with oral anticoagulation in all but the lowest risk patients. 1

## Methods

We performed a retrospective cohort analysis using data from the 2016 U.S. Agency for Healthcare Research and Quality, Healthcare Cost and Utilization Project (HCUP) Nationwide Readmissions Database (NRD). The 2016 NRD includes a full calendar year of data with hospital discharge/patient characteristics and diagnosis and procedure codes reported using the International Classification of Diseases-10th Revision-Clinical Modification or Procedural Coding System (ICD-10-CM or -PCS). The 2016 NRD contains up to 35 ICD-10-CM diagnosis codes and 15 procedural codes for each encounter. The database provides nationally representative information on hospital admissions for all patient ages and readmissions that take place in the same state as the index hospitalization. 6 Unweighted, the NRD contains data for approximately 17 million discharges each year. Weighted, it estimates roughly 35 million discharges from 27 geographically dispersed states, accounting for 57.8% of the total U.S. resident population and 56.6% of all U.S. hospitalizations.

We identified patients with an ICD-10-CM code for AF, who were ≥18 years-of-age and had an index hospitalization for a bleeding event between January 1, 2016 and November 30, 2016 (with the November 30th cut-off utilized to allow for assessment of 30-day readmission outcomes in the 2016 calendar year) ( Supplementary Table S1 ). An index bleeding-related hospitalization was defined as a patient's first hospitalization with a primary ICD-10-CM diagnosis code from the Cunningham 7 or Joos 8 algorithms indicating bleeding or select nonprimary diagnosis codes from Cunningham 7 indicating bleeding if accompanied by a transfusion-related code ( Supplementary Tables S2 S3 S4 ). Bleeding-related hospitalizations were further stratified into bleeding subtype cohorts of nontraumatic ICH, traumatic ICH, gastrointestinal, genitourinary, or other bleeding (e.g., hemopericardium, hemoperitoneum, hemarthrosis, unspecified hemorrhage).

**Table 1:** Baseline characteristics

	All bleeding-related hospitalizations *N* = 196,878 *n* (%)	Traumatic ICH *N* = 23,564 *n* (%)	Nontraumatic ICH *N* = 5,987 *n* (%)	GI bleeds *N* = 139,433 *n* (%)	GU bleeds *N* = 2,128 *n* (%)	Other *N* = 25,766 *n* (%)
Age (median, 25th, 75th percentile)	80 (72, 86)	82 (75, 88)	80 (70, 86)	79 (72, 86)	74 (61, 84)	79 (70, 86)
65–74 y	4,331 (22.0)	3,999 (17.0)	1,280 (21.4)	31,686 (22.7)	441 (20.7)	5,925 (23.0)
≥ 75 y	131,467 (66.8)	17,753 (75.3)	3,893 (65.0)	92,333 (66.2)	1,036 (48.7)	16,451 (63.8)
Sex: Female	94,438 (48.0)	10,385 (44.1)	2,909 (48.6)	67,058 (48.1)	1,130 (53.1)	12,956 (50.3)
Comorbidities						
Alcohol abuse	7,888 (4.0)	1,404 (6.0)	187 (3.1)	5,571 (4.0)	58 (2.7)	669 (2.7)
Anemia	54,835 (27.9)	4,981 (21.1)	944 (15.8)	45,614 (32.7)	503 (23.7)	2,793 (10.8)
Heart failure	76,967 (39.1)	7,004 (29.7)	1,539 (25.7)	55,974 (40.1)	736 (34.6)	11,715 (45.5)
Thrombocytopenia	14,683 (7.5)	2,374 (10.1)	387 (6.5)	10,040 (7.2)	122 (5.7)	1,760 (6.8)
Coagulopathy	24,019 (12.2)	3,548 (15.1)	668 (11.2)	16,408 (11.8)	232 (10.9)	3,163 (12.3)
Diabetes	66,968 (34.0)	7,034 (29.8)	2,061 (34.4)	47,966 (34.4)	652 (30.6)	9,256 (35.7)
Hypertension	161,769 (82.2)	19,169 (81.3)	5,279 (88.2)	115,101 (82.5)	1,281 (60.2)	20,939 (81.3)
Hypothyroidism	40,431 (20.5)	4,933 (20.9)	1,088 (18.2)	28,510 (20.4)	353 (16.6)	5,547 (21.5)
Liver disease	10,775 (5.5)	707 (3.0)	113 (1.9)	8,588 (6.2)	89 (4.2)	1,277 (5.0)
Metastatic cancer	4,253 (2.2)	316 (1.3)	162 (2.7)	3,102 (2.2)	66 (3.1)	607 (2.4)
Obesity	25,696 (13.1)	1,488 (6.3)	649 (10.8)	19,406 (13.9)	374 (17.6)	3,778 (14.7)
Peripheral vascular disease	25,607 (13.0)	2,349 (10.0)	618 (10.3)	18,979 (13.6)	278 (13.1)	3,383 (13.1)
Ischemic stroke	1,880 (1.0)	558 (2.4)	386 (6.5)	818 (0.6)	16 (0.8)	1,102 (0.4)
Renal impairment	65,084 (33.1)	5,541 (23.5)	1,320 (22.1)	48,112 (34.5)	424 (19.9)	9,687 (37.7)
CKD stage 3 or worse	49,498 (25.1)	3,871 (16.4)	960 (16.0)	37,007 (26.5)	322 (15.2)	7,338 (28.5)
Solid tumors	5,971 (3.0)	398 (1.7)	145 (2.4)	4,293 (3.1)	123 (5.8)	1,012 (3.9)
Lymphoma	2,005 (1.0)	148 (0.6)	43 (0.7)	1,486 (1.1)	18 (0.9)	311 (1.2)
Peptic ulcer disease without bleeding	3,554 (1.8)	149 (0.6)	41 (0.7)	1,921 (1.4)	17 (0.8)	1,425 (5.5)
CHA2DS2VASc score (median, 25th, 75th percentile)	4 (3,5)	4 (3,5)	4 (3,5)	4 (3,5)	3 (2,4)	4 (3,5)
CHA2DS2VASc ≥2	187,233 (95.1)	22,390 (95.0)	5,685 (94.9)	132,768 (95.2)	1,830 (86.0)	24,561 (95.3)
Modified HASBLED score (median, 25th, 75th percentile)	3 (3,4)	3 (3,4)	3 (3,3)	3 (3,4)	3 (2,3)	3 (3,4)
Modified HASBLED ≥3	161,792 (82.2)	19,368 (82.2)	4,982 (83.2)	115,384 (82.8)	1,269 (59.6)	20,789 (80.7)

Abbreviations: CKD, chronic kidney disease; GI, gastrointestinal; GU, genitourinary; ICH, intracranial hemorrhage.

**Table 2:** Crude incidence of outcomes in patients experiencing a bleeding-related hospitalization

	All bleeding-related hospitalizations *N* = 196,878 *n* (%)	Traumatic ICH *N * = 23,564 *n* (%)	Nontraumatic ICH *N * = 5,987 *n* (%)	GI bleeds *N * = 139,433 *n* (%)	GU bleeds *N * = 2,128 *n* (%)	Other *N* = 25,766 *n* (%)
In-hospital mortality	9,720 (4.9)	2,921 (12.4)	1,755 (29.3)	4,503 (3.2)	26 (1.2)	515 (2.0)
Index hospital length-of-stay, days, mean ± SD (median, 25%, 75% range)	5.45 ± 6.31 [4 (2, 6)]	7.77 ± 9.93 [5 (3, 9)]	6.95 ± 8.87 [4 (2, 9)]	5.19 ± 5.46 [4 (2, 6)]	4.30 ± 4.20 [3 (2, 5)]	4.48 ± 5.13 [3 (2, 5)]
Other discharge disposition						
Routine discharge or destination unknown	98,104 (49.8)	5,402 (22.9)	983 (16.4)	77,212 (55.4)	1,384 (65.0)	13150 (51.0)
Short-term hospital	1,826 (0.9)	280 (1.2)	117 (2.0)	1,173 (0.8)	18 (0.8)	238 (0.9)
Other: SNF, ICF, another type of facility	50,035 (25.4)	10,533 (44.7)	2,405 (40.2)	30,186 (21.6)	342 (16.1)	6569 (25.5)
Home health care	35,756 (18.2)	4,253 (18.0)	705 (11.8)	25,363 (18.2)	361 (17.0)	5074 (19.7)
Against medical advice	1,360 (0.7)	155 (0.7)	23 (0.4)	946 (0.7)	22 (1.0)	215 (0.8)
Readmission for any cause within 30 d of hospital discharge and within the 2016 calendar year a	35,899 (18.2)	3,703 (15.7)	683 (11.4)	25,869 (18.6)	407 (19.1)	5236 (20.3)
30-d readmission for a subsequent major bleeding a	7,383 (3.8)	789 (3.3)	48 (0.8)	5,690 (4.1)	36 (1.7)	820 (3.2)
30-d readmission for thrombotic event (ischemic stroke, MI, DVT, PE] a	1,964 (1.0)	235 (1.0)	108 (1.8)	1,348 (1.0)	17 (0.8)	255 (1.0)

Abbreviations: DVT, deep vein thrombosis; GI, gastrointestinal; GU, genitourinary; ICF, intermediate care facility; ICH, intracranial hemorrhage; MI, myocardial infarction; PE, pulmonary embolism; SD, standard deviation; SNF, skilled nursing facility.

a Percentage based on number of patients surviving the index hospitalization.

**Table 3:** Crude and adjusted odds ratios for outcomes stratified on bleed type

	In-hospital mortality		Post-discharge out-of-hospital care		Index hospital length-of-stay	
	Crude OR	Adjusted OR	Crude OR	Adjusted OR	Mean day(s) difference	Adjusted mean day(s) difference
Gastrointestinal	Referent	Referent	Referent	Referent	Referent	Referent
Traumatic ICH	4.24 (4.04, 4.45)	3.99 (3.79, 4.19)	2.92 (2.84, 3.01)	2.92 (2.83, 3.01)	2.68 (2.63, 2.73)	8.31 (8.03, 8.60)
Nontraumatic ICH	12.42 (11.66, 13.23)	13.09 (12.24, 13.99)	2.51 (2.38, 2.64)	2.74 (2.59, 2.90)	2.15 (2.03, 2.28)	6.27 (5.97, 6.57)
Genitourinary	0.38 (0.26, 0.55)	0.37 (0.25, 0.55)	0.70 (0.63, 0.79)	0.86 (0.77, 0.97)	−0.89 (−1.00, −0.76)	−2.84 (−2.91, −2.76)
Other	0.61 (0.56, 0.67)	0.58 (0.53, 0.64)	1.24 (1.20, 1.28)	1.30 (1.26, 1.34)	−0.70 (−0.72, −0.68)	−2.06 (−2.11, −2.01)

Abbreviations: CI, confidence interval; ICH, intracranial hemorrhage; OR, odds ratio.

Clinical outcomes evaluated in this study included the rate of bleeding-related hospitalizations (per 100,000 adult persons, assuming 244,945,724 adults in the United States in 2016 9 ), the proportion of patients dying prior to discharge (inpatient mortality), index hospitalization length-of-stay (LOS), the proportion requiring post-discharge out-of-home care at an intermediate care facility (ICF), inpatient rehabilitation facility or skilled nursing facility, and the proportion of patients surviving to discharge readmitted for any cause, subsequent bleeding or a thrombotic event (ischemic stroke, myocardial infarction, or venous thromboembolism) within 30-days of the index hospital discharge (and within the 2016 calendar year). A sensitivity analysis was also performed whereby we restricted eligible index bleeding-related hospitalizations to those patients having an ICD-10-CM diagnosis code indicating an antithrombotic-related bleeding event (D68.32, T45.515x, or T45.525x).

Baseline patient characteristics and outcomes were analyzed using descriptive statistics. Categorical data were presented as percentages, while continuous data were summarized as means ± standard deviation and/or medians with 25%, 75% ranges. We evaluated both absolute and relative associations between bleeding-related hospitalization types (i.e., traumatic and nontraumatic ICH, gastrointestinal, genitourinary, and other bleeding) and outcomes. We assessed binary study outcomes (inpatient mortality and need for post-discharge out-of-home care) with and without adjustment for age (continuous), sex, history of stroke, thrombocytopenia, anemia, heart failure, cancer, coagulopathy, obesity, alcohol abuse, hypertension, chronic kidney disease, and diabetes (all binary) using multivariable logistic regression. Results were reported as crude and adjusted odds ratios (ORs), respectively, with 95% confidence intervals (CIs). Generalized linear regression (using a gamma-distributed error and log-link) was used to evaluate associations between bleeding-related hospitalization types and the continuous outcome of index hospitalization LOS with and without adjustment for baseline demographics and comorbidities (same covariates as for binary outcomes). Results on generalized linear regression were reported as the crude and adjusted absolute mean differences with 95% CIs. For all regression analyses, gastrointestinal bleeding was set as referent (as it was the most common cause of bleeding-related hospitalizations). All data management and statistical analyses were performed using SAS version 9.4 (SAS Institute Inc., Cary, North Carolina, United States) and IBM SPSS Statistics version 26.0 (IBM Corp, Armonk, New York, United States).

Since data were de-identified and in compliance with the Health Insurance Portability and Accountability Act (HIPAA) of 1996 to preserve participant anonymity and confidentiality, our study was deemed exempt from Institutional Review Board oversight. The preparation of the study report was done in accordance with the RECORD (REporting of studies Conducted using Observational Routinely collected health Data) statement. 10

## Results

**Fig. 1:**
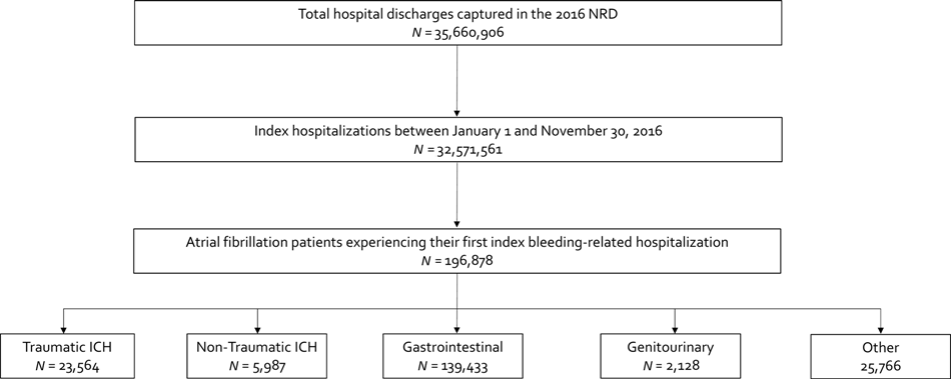
Flow diagram of patient selection.

The 2016 NRD core file contained data on 35,660,906 hospitalizations; 32,571,561 occurred between January 1, 2016 and November 30, 2016. Of these, 196,878 hospitalizations (80.4 events/100,000 persons) were bleeding-related and occurred within AF patients ( Fig. 1 ). The median age of the cohort was 80 (72, 86) years. Overall and bleeding subtype incidences increased with increasing patient age ( Supplementary Fig. S1 ). The median CHA2DS2VASC score was 4 (3, 5) and modified HASBLED score was 3 (3, 4). Ninety-five percent of this AF cohort had a CHA2DS2VASc score ≥2. Gastrointestinal bleeding (70.9%) was the most frequent bleeding-related hospitalization type followed by other bleeding (13.1%), traumatic ICH (12.0%), nontraumatic ICH (3.0%), and genitourinary bleeding (1.1%) ( Table 1 ).

### Crude Incidences

Patients with ICH (traumatic and nontraumatic) experienced a high risk of in-hospital mortality (12.4 and 29.3%) and long index hospital LOS (≥6.95 days) ( Table 2 ). ICH was also associated with a substantial proportion of patients requiring out-of-home care (45.1%) as evidenced by discharge to a secondary treatment facility. Of patients experiencing a gastrointestinal bleed-related hospitalization, 3.2% died in-hospital. Index hospital LOS averaged 5.2 ± 5.5 days and 22.2% required post-discharge out-of-home care. Genitourinary and other bleeds had in-hospital mortality rates ranging from 1.2 to 2.0%, mean LOS ranging from 4.3 to 4.5 days and 16.9 to 26.4% needed post-discharge out-of-home care. Approximately 18% of patients experiencing a bleeding-related hospitalization were readmitted for any cause within 30-days of discharge, with readmission for subsequent or rebleeding occurring in 3.8% and a thrombotic event in 1.0% of patients. Among gastrointestinal bleed patients readmitted for a subsequent bleed with 30-days ( *n* = 5690), nearly all (98.9%) were for a subsequent gastrointestinal bleed.

### Adjusted Odds Ratios

Upon multivariable regression analysis, the adjusted odds for in-hospital mortality were statistically significantly higher among patients experiencing a traumatic (adjusted OR = 3.99) or nontraumatic ICH (adjusted OR = 13.09) compared with gastrointestinal bleeding ( Table 3 ). Conversely, the adjusted ORs of in-hospital mortality were significantly lower for patients with genitourinary and other bleeds (adjusted ORs = 0.37 and 0.58, respectively) versus gastrointestinal bleeding. Traumatic and nontraumatic ICHs were also associated with significant increases in index hospitalization LOS (adjusted mean differences = 8.31 days and 6.86 days) compared with gastrointestinal bleeding. Genitourinary and other bleeds were associated with significantly reduced index hospital LOS (adjusted mean differences =  − 3.17 days and −1.84 days) versus gastrointestinal bleeding. Traumatic and nontraumatic ICH and other bleeds were found to be associated with significantly increased odds of needing subsequent post-discharge out-of-home care (adjusted ORs = 3.12, 2.84, and 1.26, respectively). Genitourinary bleeds were associated with a significantly reduced odds of needing out-of-home care compared with gastrointestinal bleeding (adjusted OR = 0.70).

### Sensitivity Analysis

Upon sensitivity analysis, we identified 20,103 patients (10.2%) with a billing code indicating antithrombotic-related bleeding ( Supplementary Table S5 ). Gastrointestinal bleeding (66.5%) was the most frequent bleeding subtype in this restricted cohort, followed by other bleeding (22.5%), traumatic ICH (7.3%), nontraumatic ICH (2.2%), and genitourinary bleeding (1.5%). Crude incidence of outcomes in this sensitivity analysis cohort is depicted in Supplementary Table S6 .

## Discussion

In this large, real-world study of nearly 200,000 AF patients hospitalized for bleeds, we found that bleeding-related hospitalizations are associated with differing levels of detrimental clinical consequences across various bleed types. Gastrointestinal bleeds were identified as the underlying cause of nearly three-quarters of all bleeding-related hospitalizations in this study but was associated with less mortality and morbidity compared with ICH. While ICH was relatively uncommon, it was associated with the greatest odds of in-hospital mortality, longer LOS, and a more frequent need for post-discharge out-of-home care. Both genitourinary and other bleeds were also relatively uncommon causes of bleeding-related hospitalizations compared with gastrointestinal bleeds. They were generally associated with the least severe outcomes of all bleeding types assessed (although genitourinary bleeds were associated with the highest readmissions for any reason within 30 days).

Our data suggest that even among patients experiencing an ICH, the risk of mortality and morbidity within patients can vary. Within ICH-related hospitalizations, we found nontraumatic ICH patients had poorer outcomes compared with traumatic ICH patients, although patients with traumatic ICH had longer hospital LOS. Siddique et al 11 previously showed the 6-month risk of death and moderate-to-severe disability (according to the Glasgow Outcome Scale) to be higher in patients experiencing a nontraumatic ICH versus a traumatic ICH (39.5 vs. 25.6% for death; 49.3 vs. 39.5% for moderate-to-severe disability). Our study reinforces the findings observed in Siddique et al but also extends them by adding healthcare utilization data. Our data suggest nontraumatic ICH is associated with nearly twice the mortality rate and three times the risk of readmission for bleeding at 30 days versus traumatic ICH. Moreover, we observed modest increases in index hospital LOS with nontraumatic versus traumatic ICH. Although we were unable to assess for the presence of disability in our study, we did observe that only 24.5% of the patients with nontraumatic ICH were discharged home (with or without home health care services) compared with 40.3% of patients hospitalized for a traumatic ICH. These findings suggest ICH, in general, was associated with considerable morbidity.

Gastrointestinal bleeding was the cause of bleeding-related hospitalization in 70.8% of patients in our cohort. While billing codes potentially allow for the determination of the specific type of gastrointestinal bleeding (e.g., gastroduodenal, esophageal, upper, lower), as many as one-third of gastrointestinal bleeds are coded in a fashion that does not allow determination of even general anatomical location (e.g., ICD-10 K92.2 = “Gastrointestinal hemorrhage, unspecified”). 7 For this reason, we did not attempt to evaluate outcomes stratified by gastrointestinal bleeding type/location. Given the large number of detected bleeds in our study that were gastrointestinal in nature, we assessed the proportion of all 30-day readmissions that were due to a subsequent gastrointestinal bleed. Our data suggest that nearly all subsequent bleeding events in the index gastrointestinal bleed population, were again, gastrointestinal in nature.

While the NRD does not provide data on medications used prior to (or during) admission, ICD-10-CM billing codes specifically identifying antithrombotic-related bleeding events exist (D68.32, T45.515x, and T45.525x) and we performed a sensitivity analysis whereby we restricted the population to patients with at least one of these billing codes. A code indicating antithrombotic-related bleeding was identified in only 10.2% of the overall bleeding-related hospitalization cohort, with crude incidences of outcomes appearing generally in line with the overall cohort. A previous study by Shehab et al assessed the accuracy of ICD-10-CM codes to identify antithrombotic-related bleeding hospitalizations in Medicare fee‐for‐service beneficiaries who were hospitalized for a major bleed at one of five hospitals across three states and found they yielded only a moderate positive predictive value (71.4%) and poor sensitivity (6.8%). 12 As with the study conducted by Shehab, it is likely that the number of antithrombotic-related bleed is artificially low due to underutilization of corresponding codes. Given the high thrombotic risk nature of our study cohort (all with AF at presentation, >95% with a CHA2DS2VASc score ≥2), we would anticipate a large portion of patients were receiving an oral anticoagulant at baseline. 13 14 Data from the International Global Anticoagulant Registry in the FIELD (GARFIELD)-AF and the United States National Outcomes Registry for Better Informed Treatment of Atrial Fibrillation (ORBIT-AF) both depicted relatively high oral anticoagulant utilization, with 56 to 65% of AF patients with a CHA2DS2VASc = 1 and 70 to 87% with a CHA2DS2VASc ≥ 2 receiving oral anticoagulation. 13 This suggests the diagnosis of AF may serve as a reasonable proxy for oral anticoagulant use. However, underutilization of oral anticoagulation therapy among patients with AF is likely still suboptimal in specific cohorts. The American College of Cardiology's Practice Innovation and Clinical Excellence (PINNACLE) registry demonstrated the prevalence of oral anticoagulant use (from 2008 to 2012) did not exceed 50.6% even in patients with AF and a CHA2DS2-VASc score exceeding 4. 14 Importantly, since we limited this study to AF patients only, our findings are less generalizable to bleeding-related hospitalizations in other populations who may receive oral anticoagulation (e.g., venous thromboembolism).

Our study has limitations worthy of discussion. First, as with all retrospective administrative claims-based analyses, the influence of both selection bias (i.e., distortion in a measure of association due to a sample selection that does not accurately reflect the target population) and misclassification bias (i.e., classification of an individual, value, or attribute into a category other than that to which it belongs) on the study's internal validity need to be considered. 15 The NRD captures all inpatients discharges in 27 geographically diverse states that participate in the HCUP State Inpatient Databases. This sample is then statistically weighted to make it representative of all inpatient discharges (nonrehabilitation or long-term acute care facility) at U.S. hospitals. 5 The use of published, validated coding algorithms by Cunningham et al 7 (overall algorithm positive predictive value of 89%) and Joos et al 8 (algorithm sensitivity of 91.4% and specificity of 90.2%) to identify bleeding-related hospitalizations were utilized to reduce the risk of misclassification bias in our study and is a reasonable proxy for clinically relevant, if not major bleeding. Second, the NRD lacks data on medication use, disability assessment, diagnostic tests (e.g., endoscopy reports), and laboratory values (e.g., hematocrit/hemoglobin). As noted above, we attempted to overcome many of these limitations through the use of various billing code algorithms (likely with varying success) as well as through the use of proxy outcomes (i.e., assessment of the need for post-discharge out-of-home care in lieu of formal disability assessments or scales). Future studies using datasets with detailed pharmacy claims or drug utilization data that can overcome such limitations are warranted to confirm our findings. Subtypes of ICH were not consistently reported in the NRD therefore we were unable to contrast outcomes of intracerebral, subdural, and subarachnoid hemorrhages. Finally, the NRD only allows the identification of readmissions that occurred in the same state (and year) as the index hospitalization. Thus, our study may underestimate readmission rates. 6

## Conclusion

This large study suggests that in AF patients, bleeding-related hospitalizations were associated with a substantial burden-of-illness. Burden was notably driven by relatively rare, but severe and/or life-threatening ICH. While being the most frequently observed bleeding-related hospitalization cause, gastrointestinal bleeding was infrequently associated with in-hospital mortality or need for out-of-home post-discharge care. We found ICH to be associated with increased odds of in-hospital mortality, extended LOS, and post-discharge out-of-home care compared with gastrointestinal bleeding, while genitourinary and other bleeds appeared to be associated with the least detrimental outcomes. This study provides much needed, nationally representative and contemporary data on in-hospital and 30-day clinical outcomes of bleeding-associated hospitalizations in AF patients, both overall and by bleed subtype. These findings provide clinicians and other healthcare decision-makers needed data on absolute and relative risks of detrimental outcomes by bleeding-related hospitalization type. Further, our study emphasizes the need for continued research on bleeding risk factors and mitigation techniques to avoid bleeding-related patient hospitalizations.

## References

[1] ESC Scientific Document Group Kirchhof P Benussi S Kotecha D 2016 ESC guidelines for the management of atrial fibrillation developed in collaboration with EACTS Eur Heart J 2016 37 38 2893 2962 27567408 10.1093/eurheartj/ehw210

[2] Jakobsen M Kolodziejczyk C Klausen Fredslund E Poulsen P B Dybro L Paaske Johnsen S Costs of major intracranial, gastrointestinal and other bleeding events in patients with atrial fibrillation—a nationwide cohort study BMC Health Serv Res 2017 17 01 398 28606079 10.1186/s12913-017-2331-z PMC5469002

[3] Inohara T Xian Y Liang L Association of intracerebral hemorrhage among patients taking non-vitamin K antagonist vs vitamin K antagonist oral anticoagulants with in-hospital mortality JAMA 2018 319 05 463 473 29372247 10.1001/jama.2017.21917 PMC5839299

[4] Deitelzweig S Neuman W R Lingohr-Smith M Menges B Lin J Incremental economic burden associated with major bleeding among atrial fibrillation patients treated with factor Xa inhibitors J Med Econ 2017 20 12 1217 1223 28760063 10.1080/13696998.2017.1362412

[5] Angamo M T Chalmers L Curtain C M Bereznicki L R Adverse-drug-reaction-related hospitalisations in developed and developing countries: a review of prevalence and contributing factors Drug Saf 2016 39 09 847 857 27449638 10.1007/s40264-016-0444-7

[6] H CUP. Introduction to the HCUP Nationwide Readmissions Database (NRD). 2010–2016. Accessed November 15, 2019 at:https://www.hcup-us.ahrq.gov/db/nation/nrd/Introduction_NRD_2010-2016.jsp

[7] Cunningham A Stein C M Chung C P Daugherty J R Smalley W E Ray W A An automated database case definition for serious bleeding related to oral anticoagulant use Pharmacoepidemiol Drug Saf 2011 20 06 560 566 21387461 10.1002/pds.2109 PMC3365595

[8] Joos C Lawrence K Jones A E Johnson S A Witt D M Accuracy of ICD-10 codes for identifying hospitalizations for acute anticoagulation therapy-related bleeding events Thromb Res 2019 181 71 76 31357146 10.1016/j.thromres.2019.07.021

[9] United States Census Bureau American Fact Finder 2017. Accessed October 21, 2019 at:https://factfinder.census.gov/faces/tableservices/jsf/pages/productview.xhtml?src=bkmk

[10] RECORD Working Committee Benchimol E I Smeeth L Guttmann A The REporting of studies Conducted using Observational Routinely-collected health Data (RECORD) statement PLoS Med 2015 12 10 e1001885 26440803 10.1371/journal.pmed.1001885 PMC4595218

[11] Siddique M S Gregson B A Fernandes H M Comparative study of traumatic and spontaneous intracerebral hemorrhage J Neurosurg 2002 96 01 86 89 11794609 10.3171/jns.2002.96.1.0086

[12] Shehab N Ziemba R Campbell K N Assessment of ICD-10-CM code assignment validity for case finding of outpatient anticoagulant-related bleeding among Medicare beneficiaries Pharmacoepidemiol Drug Saf 2019 28 07 951 964 31144403 10.1002/pds.4783 PMC13086182

[13] GARFIELD-AF ORBIT-AF Investigators Steinberg B A Gao H Shrader P International trends in clinical characteristics and oral anticoagulation treatment for patients with atrial fibrillation: results from the GARFIELD-AF, ORBIT-AF I, and ORBIT-AF II registries Am Heart J 2017 194 132 140 29223431 10.1016/j.ahj.2017.08.011

[14] Hsu J C Maddox T M Kennedy K F Oral anticoagulant therapy prescriptions in patients with atrial fibrillation across the spectrum of stroke risk: insights from the NCDR PINNACLE Registry JAMA Cardiol 2016 1 01 55 62 27437655 10.1001/jamacardio.2015.0374

[15] Gandhi S K Salmon W Kong S X Zhao S Z Administrative databases and outcomes assessment: an overview of issues and potential utility J Manag Care Spec Pharm 1999 5 215 222

