# Supplementary material for: Burden-of-Illness Associated with Bleeding-Related Hospitalizations in Atrial Fibrillation Patients: Findings from the Nationwide Readmission Database
Source: TH Open. 2020 Sep 11;4(3):e211–7. doi: 10.1055/s-0040-1716549 (PMC7553795; doi:10.1055/s-0040-1716549)
Supplement: Supplementary file 1 — Supplementary Material [file 10-1055-s-0040-1716549-s2030012.pdf]

Supplementary Table S1 List of baseline and outcome variable codes

Variable	Code(s)	Code description
Atrial fibrillation	I48	ICD-10 diagnosis
Thrombocytopenia	D69.1, D69.3, D69.4, D69.41, D69.42, D69.49, D69.59, D69.6, D75.82	ICD-10 diagnosis
Ischemic stroke	I63	ICD-10 diagnosis
Chronic kidney disease stage 3 or worse	N18.3, N18.4, N18.5, N18.6	ICD-10 diagnosis
Deep vein thrombosis	I80.00, I80.01, I80.02, I80.03, I80.10, I80.11, I80.12, I80.13, I80.201, I80.202, I80.203, I80.209, I80.211, I80.212, I80.213, I80.219, I80.221, I80.222, I80.223, I80.229, I80.231, I80.232, I80.233, I80.239, I80.291, I80.292, I80.293, I80.299, I80.3, I80.8, I80.9, I81, I82.xxx	ICD-10 diagnosis
Pulmonary embolism	I26.01, I26.02, I26.09, I26.90, I26.92, I26.99	ICD-10 diagnosis
Myocardial infarction	I21, I21.0, I21.01, I21.02, I21.09, I21.1, I21.11, I21.19, I21.2, I21.21, I21.29, I21.3, I21.4, I22, I22.0, I22.1, I22.2, I22.8, I22.9, I23, I23.0, I23.1, I23.2, I23.3, I23.4, I23.5, I23.6, I23.7, I23.8, I25.2	ICD-10 diagnosis
Alcohol abuse	Per the Elixhauser algorithm for comorbidity measures	ICD-10 diagnosis
Anemia	Per the Elixhauser algorithm for comorbidity measures	ICD-10 diagnosis
Heart failure	Per the Elixhauser algorithm for comorbidity measures	ICD-10 diagnosis
Coagulopathy	Per the Elixhauser algorithm for comorbidity measures	ICD-10 diagnosis
Diabetes	Per the Elixhauser algorithm for comorbidity measures	ICD-10 diagnosis
Hypertension	Per the Elixhauser algorithm for comorbidity measures	ICD-10 diagnosis
Hypothyroidism	Per the Elixhauser algorithm for comorbidity measures	ICD-10 diagnosis
Liver disease	Per the Elixhauser algorithm for comorbidity measures	ICD-10 diagnosis
Metastatic cancer	Per the Elixhauser algorithm for comorbidity measures	ICD-10 diagnosis
Obesity	Per the Elixhauser algorithm for comorbidity measures	ICD-10 diagnosis
Peripheral vascular disease	Per the Elixhauser algorithm for comorbidity measures	ICD-10 diagnosis
Renal impairment	Per the Elixhauser algorithm for comorbidity measures	ICD-10 diagnosis
Solid tumors	Per the Elixhauser algorithm for comorbidity measures	ICD-10 diagnosis
Lymphoma	Per the Elixhauser algorithm for comorbidity measures	ICD-10 diagnosis
Peptic ulcer disease (without bleeding)	Per the Elixhauser algorithm for comorbidity measures	ICD-10 diagnosis

Abbreviation: ICD-10, International Classification of Diseases-10th Revision.[1]

Supplementary Table S2 List of primary bleed codes

Variable	Code(s)	Code description
Gastroduodenal site bleed	K250,K5660,K252,K254,K256,K260,K262,K264,K266,K270,K272,K274,K276, K280,K282,K284,K286,K2901,K2941,K2961,K2921,K2971,K2981,K31811, K2931,K2951,K2991	ICD-10 diagnosis
Esophageal site bleed	I8501,I8511,I8510,K226,K228,K2211	ICD-10 diagnosis
Upper gastrointestinal bleed, unspecified	K920	ICD-10 diagnosis
Lower gastrointestinal site bleed	K648,K644,K5711,K5713,K5731,K5733,K661,K625,K5521,K50011,K50111, K50811,K50911,K51011,K51211,K51311,K51411,K51511,K51811,K51911, K5701,K5721,K5741,K5751,K5753,K5781,K5791,K5793	ICD-10 diagnosis
Gastrointestinal site bleed, unspecified	K921,K922	ICD-10 diagnosis
Genitourinary site	N280,R319,N898,N920,N921,N897,N925,N93,N938,N939,O717,R31,R319	ICD-10 diagnosis
Intracranial site bleed	I609,I619,I621,I629	ICD-10 diagnosis
Other site bleed	I312,R58,K661,M2500,M25019,M25029,M25039,M25049,M25059,M25069, M25073,M25076,M2508,R040,R041,R042,H05239,H2103,H31309,H31319, H31329,H31419,H3560,H35739,H4313,H44819,H47029,H61129,M7981, N837,R0489,R049	ICD-10 diagnosis
Traumatic intracranial bleed	S06359A,S06360A,S064 × 0A,S064 × 0D,S065 × 0A,S065 × 0D,S065 × 1A, S065 × 9A,S065 × 9D,S066 × 0A,S066 × 0D,S066 × 1A,S066 × 9A,S066 × 9D, S0634,S06370A,S06371A,S06372A,S06373A,S06374A,S06375A,S06376A, S06377A,S06378A,S06379A,S063780A,S063781A,S063782A,S063783A, S063784A,S063785A,S063786A,S063787A,S063788A,S063789A,S06380A, S06381A,S06382A,S06383A,S06384A,S06385A,S06386A,S06387A,S06388A, S06389A,S065 × 0A,S065 × 1A,S065 × 2A,S065 × 3A,S065 × 4A,S065 × 5A, S065 × 6A,S065 × 7A,S065 × 8A,S065 × 9A	ICD-10 diagnosis

Abbreviation: ICD-10, International Classification of Diseases-10th Revision.

Supplementary Table S3 List of nonprimary bleed codes

Variable	Code(s)	Code description
Gastroduodenal site bleed	K251,K253,K255,K257,K259,K261,K263,K265,K267, K269,K271,K273,K275,K277,K279,K281,K283,K285, K287,K289,K2900,K2940,K2950,K2960,K2920,K2970,K2990,K2980	ICD-10 diagnosis
Lower gastrointestinal site bleed	K648,K644,K645,K649,K5710,K5712,K5730,K5732	ICD-10 diagnosis
Esophageal site bleed	K209	ICD-10 diagnosis
Unspecified site bleed	D500,D62,D649,R791	ICD-10 diagnosis

Abbreviation: ICD-10, International Classification of Diseases-10th Revision.

Supplementary Table S4 List of blood transfusion codes

Variable	Code(s)	Code description
Blood transfusion	30230AZ,30230G0,30230G2,30230G3,30230G4,30230H0,30230H1,30230J0,30230J1, 30230K0,30230K1,30230L0,30230L1,30230M0,30230M1,30230N0,30230N1,30230P0, 30230P1,30230Q0,30230Q1,30230R0,30230R1,30230S0,30230S1,30230T0,30230T1, 30230V0,30230V1,30230W0,30230W1,30230 × 0,30230 × 2,30230 × 3,30230 × 4, 30230Y0,30230Y2,30230Y3,30230Y4,30233AZ,30233G0,30233G2,30233G3,30233G4, 30233H0,30233H1,30233J0,30233J1,30233K0,30233K1,30233L0,30233L1,30233M0, 30233M1,30233N0,30233N1,30233P0,30233P1,30233Q0,30233Q1,30233R0,30233R1, 30233S0,30233S1,30233T0,30233T1,30233V0,30233V1,30233W0,30233W1, 30233 × 0,30233 × 2,30233 × 3,30233 × 4,30233Y0,30233Y2,30233Y3,30233Y4, 30240AZ,30240G0,30240G2,30240G3,30240G4,30240H0,30240H1,30240J0,30240J1, 30240K0,30240K1,30240L0,30240L1,30240M0,30240M1,30240N0,30240N1,30240P0, 30240P1,30240Q0,30240Q1,30240R0,30240R1,30240S0,30240S1,30240T0,30240T1, 30240V0,30240V1,30240W0,30240W1,30240 × 0,30240 × 2,30240 × 3,30240 × 4, 30240Y0,30240Y2,30240Y3,30240Y4,30243AZ,30243G0,30243G2,30243G3,30243G4, 30243H0,30243H1,30243J0,30243J1,30243K0,30243K1,30243L0,30243L1,30243M0, 30243M1,30243N0,30243N1,30243P0,30243P1,30243Q0,30243Q1,30243R0,30243R1, 30243S0,30243S1,30243T0,30243T1,30243V0,30243V1,30243W0,30243W1,30243 × 0,30243 × 2,30243 × 3,30243 × 4,30243Y0,30243Y2,30243Y3,30243Y4,30250G0, 30250G1,30250H0,30250H1,30250J0,30250J1,30250K0,30250K1,30250L0,30250L1, 30250M0,30250M1,30250N0,30250N1,30250P0,30250P1,30250Q0,30250Q1,30250R0, 30250R1,30250S0,30250S1,30250T0,30250T1,30250V0,30250V1,30250W0,30250W1, 30250 × 0,30250 × 1,30250Y0,30250Y1,30253G0,30253G1,30253H0,30253H1, 30253J0,30253J1,30253K0,30253K1,30253L0,30253L1,30253M0,30253M1,30253N0, 30253N1,30253P0,30253P1,30253Q0,30253Q1,30253R0,30253R1,30253S0,30253S1, 30253T0,30253T1,30253V0,30253V1,30253W0,30253W1,30253 × 0,30253 × 1,30253 Y0,30253Y1,30260G0,30260G1,30260H0,30260H1,30260J0,30260J1,30260K0, 30260K1,30260L0,30260L1,30260M0,30260M1,30260N0,30260N1,30260P0,30260P1, 30260Q0,30260Q1,30260R0,30260R1,30260S0,30260S1,30260T0,30260T1,30260V0, 30260V1,30260W0,30260W1,30260 × 0,30260 × 1,30260Y0,30260Y1,30263G0,30263-G1,30263H0,30263H1,30263J0,30263J1,30263K0,30263K1,30263L0,30263L1, 30263M0,30263M1,30263N0,30263N1,30263P0,30263P1,30263Q0,30263Q1,30263R0, 30263R1,30263S0,30263S1,30263T0,30263T1,30263V0,30263V1,30263W0,30263W1, 30263 × 0,30263 × 1,30263Y0,30263Y1,30273H1,30273J1,30273K1,30273L1,30273M1, 30273N1,30273P1,30273Q1,30273R1,30273S1,30273T1,30273V1,30273W1,30277H1, 30277J1,30277K1,30277L1,30277M1,30277N1,30277P1,30277Q1,30277R1,30277S1, 30277T1,30277V1,30277W1,30280B1,30283B1	ICD-10 procedure

Supplementary Table S5 Baseline characteristics of patients with a billing code indicating an antithrombotic-related bleed

	**Any antithrombotic-related bleed** ***N*** **= 20,103** ***n*** **(%)**
Age (median, 25th, 75th percentile)	80 (72, 86)
65–74 y	4,404 (21.9)
≥ 75 y	13,637 (67.8)
Sex: Female	9,644 (48.0)
Comorbidities	
Alcohol abuse	624 (3.1)
Anemia	5,204 (25.9)
Heart failure	8,692 (43.2)
Thrombocytopenia	1,444 (7.2)
Coagulopathy	5,769 (28.7)
Diabetes	6,924 (34.4)
Hypertension	16,620 (82.7)
Hypothyroidism	4,003 (19.9)
Liver disease	808 (4.0)
Metastatic cancer	412 (2.0)
Obesity	2,903 (14.4)
Peripheral vascular disease	2,590 (12.9)
Ischemic stroke	121 (0.6)
Renal impairment	7,176 (35.7)
CKD Stage 3 or worse	5,478 (27.3)
Solid tumors	567 (2.8)
Lymphoma	162 (0.8)
Peptic ulcer disease without bleeding)	262 (1.3)
CHA2DS2VASc score (median, 25th, 75th percentile)	4 (3, 5)
CHA2DS2VASc ≥2	19,337 (96.2)
Modified HASBLED score (median, 25th, 75th percentile)	3 (3, 4)
Modified HASBLED ≥3	16,633 (82.7)

Abbreviations: CKD, chronic kidney disease; GI, gastrointestinal; GU, genitourinary; ICH, intracranial hemorrhage.

Supplementary Table S6 Crude incidence of outcomes in patients experiencing an antithrombotic-related bleed

	**Any antithrombotic-related bleed** ***N*** **= 20,103** ***n*** **(%)**
In-hospital mortality (20)	851 (4.2)
Index hospital length of stay, days, mean ± SD [median, 25%, 75% range]	5.34 ± 5.61 [4 (3, 6)]
Other discharge disposition	
Routine discharge or destination unknown	9,771 (48.6)
Short-term hospital	156 (0.8)
Other: SNF, ICF, another type of facility	5,266 (26.2)
Home health care	3,939 (19.6)
Against medical advice	106 (0.5)
Readmission for any cause within 30 d of hospital discharge and within the 2016 calendar year[a]	3,533 (17.6)
30-d readmission for a subsequent major bleeding[a]	569 (2.8)
30-d readmission for thrombotic event [ischemic stroke, MI, DVT, PE][a]	220 (1.1)

Abbreviations: DVT, deep vein thrombosis; GI, gastrointestinal; GU, genitourinary; ICF, intermediate care facility; ICH, intracranial hemorrhage; MI, myocardial infarction; PE, pulmonary embolism; SNF, skilled nursing facility.

[a]Percentage based on number of patients surviving the index hospitalization.

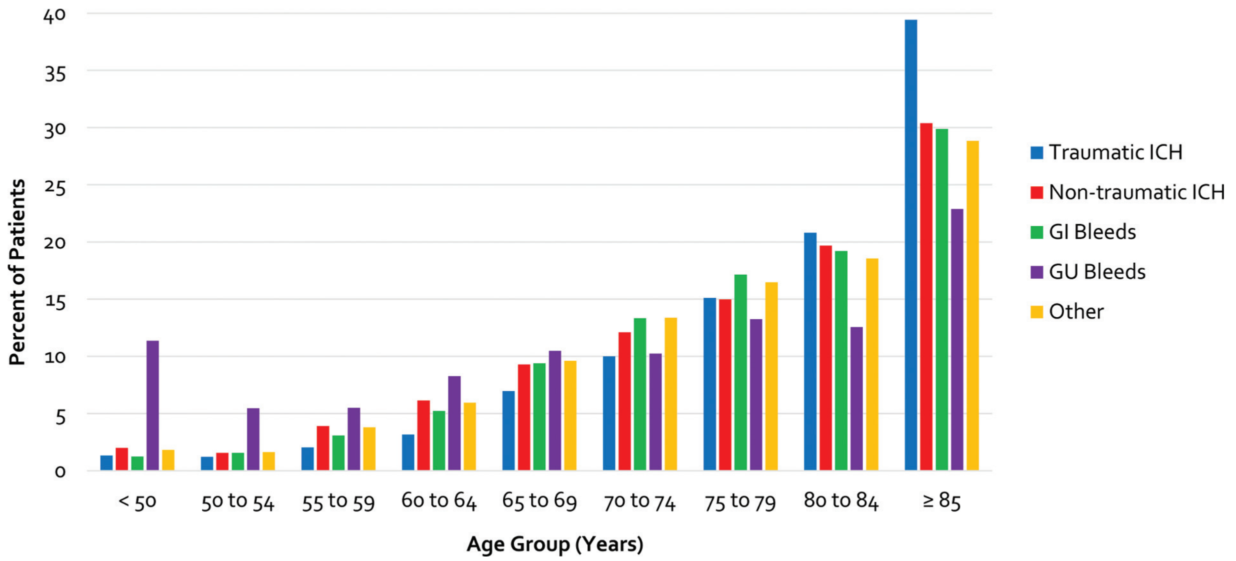

Supplementary Fig. S1 Histogram of incident bleed type by age group. ICH, intracranial hemorrhage; GI, gastrointestinal; GU, genitourinary.

Reference

1 Elixhauser A, Steiner C, Harris DR, Coffey RM. Comorbidity measures for use with administrative data. Med Care 1998; 36:8–27
